# Assessment of safety, efficacy, and dosimetry of a novel 18-kDa translocator protein ligand, [^11^C]CB184, in healthy human volunteers

**DOI:** 10.1186/s13550-017-0271-6

**Published:** 2017-03-23

**Authors:** Muneyuki Sakata, Kenji Ishibashi, Masamichi Imai, Kei Wagatsuma, Kenji Ishii, Kentaro Hatano, Kiichi Ishiwata, Jun Toyohara

**Affiliations:** 1 Research Team for Neuroimaging, Tokyo Metropolitan Institute of Gerontology, 35-2 Sakae-cho, Itabashi-ku, 173-0015 Tokyo, Japan; 2 Department of Radiology, Toranomon Hospital, Tokyo, Japan; 3 Faculty of Medicine, University of Tsukuba, Tsukuba, Japan; 4 Institute of Cyclotron and Drug Discovery Research, Southern TOHOKU Research Institute for Neuroscience, Koriyama, Japan; 5 Department of Biofunctional Imaging, Fukushima Medical University, Fukushima, Japan

## Abstract

**Background:**

*N*,*N*-di-*n*-propyl-2-[2-(4-[^11^C]methoxyphenyl)-6,8-dichloroimidazol[1,2-a]pyridine-3-yl]acetamide ([^11^C]CB184) is a novel selective radioligand for the 18-kD translocator protein (TSPO), which is upregulated in activated microglia in the brain, and may be useful in positron emission tomography (PET). We examined the safety, radiation dosimetry, and initial brain imaging with [^11^C]CB184 in healthy human volunteers.

**Results:**

Dynamic [^11^C]CB184 PET scans (90 min) were performed in five healthy male subjects. During the scan, arterial blood was sampled at various time intervals, and the fraction of the parent compound in plasma was determined with high-performance liquid chromatography. No serious adverse events occurred in any of the subjects throughout the study period. [^11^C]CB184 was metabolized in the periphery: 36.7% ± 5.7% of the radioactivity in plasma was detected as the unchanged form after 60 min. The total distribution volume (*V*_T_) was estimated with a two-tissue compartment model. The *V*_T_ of [^11^C]CB184 was highest in the thalamus (5.1 ± 0.4), followed by the cerebellar cortex (4.4 ± 0.2), and others. Although regional differences were small, the observed [^11^C]CB184 binding pattern was consistent with the TSPO distribution in the normal human brain. Radiation dosimetry was determined in three healthy male subjects using a serial whole-body PET scan acquired over 2 h after [^11^C]CB184 injection. [^11^C]CB184 PET demonstrated high uptake in the gallbladder at a later time (>60 min). In urine obtained approximately 100 min post-injection, 0.3% of the total injected radioactivity was recovered, indicating hepatobiliary excretion of radioactivity. The absorbed dose (μGy/MBq) was highest in the kidneys (21.0 ± 0.5) followed by the lungs (16.8 ± 2.7), spleen (16.6 ± 6.6), and pancreas (16.5 ± 2.2). The estimated effective dose for [^11^C]CB184 was 5.9 ± 0.6 μSv/MBq.

**Conclusions:**

This initial evaluation indicated that [^11^C]CB184 is feasible for imaging of TSPO in the brain.

**Electronic supplementary material:**

The online version of this article (doi:10.1186/s13550-017-0271-6) contains supplementary material, which is available to authorized users.

## Background

Microglia are the resident macrophages in the central nervous system (CNS) and are activated in response to pathological events such as infectious disease, inflammation, neuronal injury, ischemia, brain tumors, and neurodegenerative and neuropsychiatric disorders [1–4]. Therefore, activation of microglia in response to brain insults could be used as a disease marker for multiple CNS disorders.

Microglia express the 18-kDa translocator protein (TSPO), formerly called the peripheral benzodiazepine receptor, in the outer mitochondrial membrane [5]. In the healthy brain, the expression level of TSPO in microglia is low. When microglia are activated in response to brain injury, TSPO expression is markedly upregulated [6]. Therefore, overexpression of TSPO is considered a marker of activated microglia. Thus, radiolabeled TSPO ligands have been developed as in vivo imaging probes for detecting activated microglia with positron emission tomography (PET) in lesioned areas of the brain. This strategy may be useful for understanding the pathogenesis of various CNS disorders and assessing the efficacy of treatment for neuroinflammation.

The prototype compound, (*R*)-*N*-(sec-butyl)-4-(2-chlorophenyl)-*N*-^11^C-methyl-2-naphthamide [(*R*)-[^11^C]PK11195], has been widely used as a PET tracer for imaging TSPO expression in humans [1, 5, 7, 8]. However, (*R*)-[^11^C]PK11195 has several limitations, including its low signal-to-noise ratio, highly variable kinetics, and apparent lack of sensitivity for detecting low levels of microglial activation. These drawbacks of (*R*)-[^11^C]PK11195 are mainly due to its low binding affinity to TSPO and high lipophilicity, which result in high levels of nonspecific binding and extensive binding to plasma proteins [9]. Thus, several chemically diverse radioligands with high affinity for TSPO and lower lipophilicity have been developed as alternatives to (*R*)-[^11^C]PK11195 [9, 10] and evaluated in humans [11–16]. These ligands include phenoxy arylamides (e.g., [^11^C]DAA1106 [11], [^11^C]PBR06 [12], [^11^C]PBR28 [13], and [^18^F]FEPPA [14]) and pyrazolopyrimidines (e.g., [^11^C]DPA-713 [15] and [^18^F]DPA-714 [16]). However, clinical trials with these new TSPO ligands showed variable results in patients [17, 18], indicating the importance of developing a variety of new radiotracers with appropriate sensitivity and specificity. So another candidate of new TSPO ligand with different structural class should be considered, which may avoid individual difference of TSPO binding.

Recently, Hatano et al. developed the imidazopyridineacetamide, *N*,*N*-di-*n*-propyl-2-[2-(4-[^11^C]methoxyphenyl)-6,8-dichloroimidazol[1,2-a]pyridine-3-yl]acetamide ([^11^C]CB184), as a novel selective radioligand for TSPO [19]. The affinity of CB184 for TSPO is 7.9 times higher than that of (*R*)-PK11195 (*K*_i_ = 0.54 and 4.27 nM, respectively). The relative TSPO binding affinity of CB184 (7.9-fold) to (*R*)-PK11195 is higher than those of PBR28 (2–5-folds) [13], DPA-713 (2-fold) [15], and DPA-714 (1.3-fold) [16]. In addition, CB184 has lower lipophilicity than (*R*)-PK11195 (logP = 2.06 and 2.54, respectively). Preclinical efficacy studies showed that the regional uptake of [^11^C]CB184 into inflamed areas is comparable to uptake of (*R*)-[^11^C]PK11195 in the 6-hydroxydopamine-injured striatum [19] but higher in the herpes encephalitis rat model [20]. Furthermore, the radiosynthesis of [^11^C]CB184 was straightforward with high production yield [19] that will meet the GMP standards for human use.

Very recently, Toyohara et al. conducted preclinical safety, radiation dosimetry, and the first PET imaging studies of [^11^C]CB184 in a normal volunteer [21]. The radiation-absorbed dose estimated from murine distribution data is highest in the lung but similar in magnitude to most other ^11^C-labeled PET tracers [22]. The absence of any abnormalities in rats in the acute toxicity test and the absence of mutagenicity of CB184 together demonstrated the clinical suitability of [^11^C]CB184 for use in PET studies in humans. Furthermore, the first brain imaging with PET following administration of [^11^C]CB184 was performed safely in a normal human volunteer. These findings prompted us to further undertake initial evaluation of [^11^C]CB184 in more human subjects in a phase 1 study. Here, we report the safety, radiation dosimetry, and initial brain imaging with [^11^C]CB184 in healthy human subjects.

## Results

### Safety monitoring

The mean ± SD of the administered mass of [^11^C]CB184 was 4.9 ± 2.1 μg (range, 2.7–8.1 μg). Administration of [^11^C]CB184 was well tolerated by all subjects. No adverse or clinically detectable pharmacologic effects were seen in any of the eight subjects. No clinically important trends indicative of a safety concern were noted for laboratory parameters, vital signs, or electrocardiogram parameters.

### Brain PET scanning

**Fig. 1:**
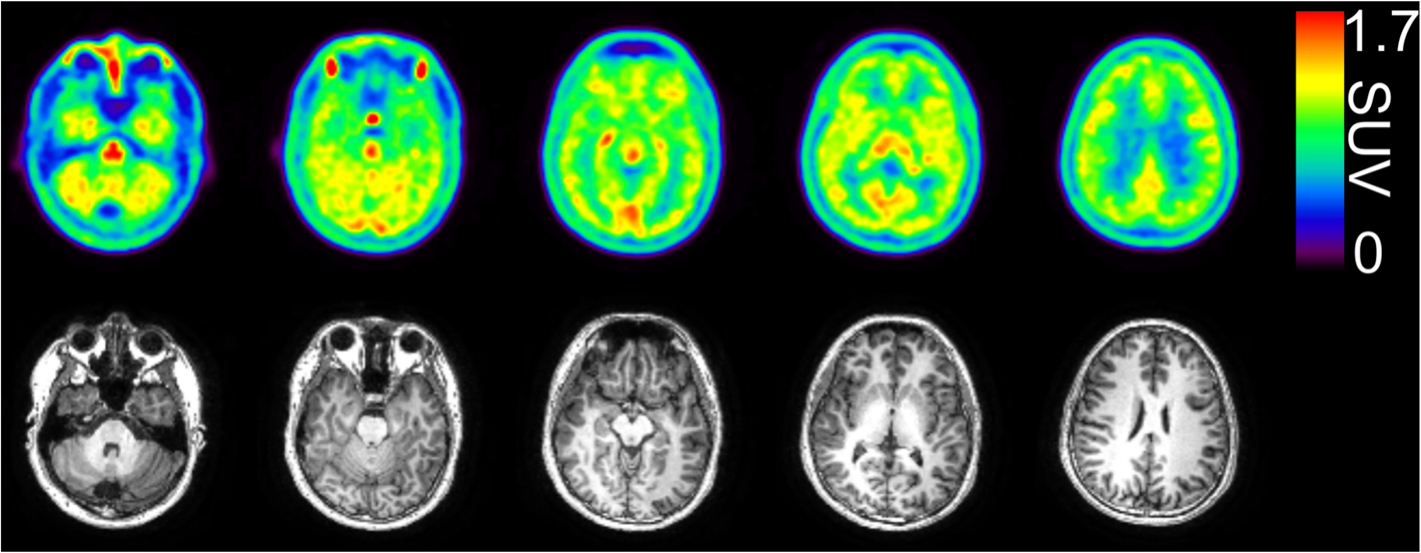
Representative magnetic resonance and static images of [^11^C]CB184 PET obtained from a 28-year-old male subject. (*Upper*) [^11^C]CB184 PET images (SUV summed 40–60 min). (*Lower*) Magnetic resonance images. PET images were smoothed with a Gaussian filter of 4 mm in FWHM

Figure 1 shows the representative static [^11^C]CB184 images (upper row) and magnetic resonance imaging (MRI) (lower row) of the corresponding slices obtained from a typical subject. The tracer was homogeneously distributed in the brain gray matter regions.

**Fig. 2:**
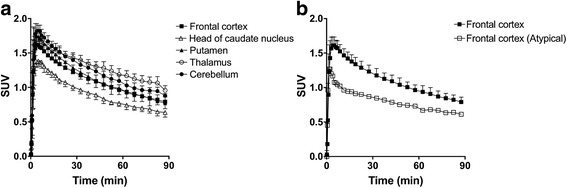
Mean decay-corrected TACs of five brain regions after intravenous injection of [^11^C]CB184 into typical human subjects (**a**). Comparison of TACs in the frontal cortex for typical subjects and an atypical subject (**b**). Data for typical subjects represent the mean ± SD of four subjects

Figure 2a shows the mean time activity curves (TACs) in five brain regions of typical subjects (*n* = 4) after intravenous injection of [^11^C]CB184. Radioactivity in all gray matter regions peaked at about 5 min. In contrast, one atypical subject showed faster brain kinetics, resulting in lower brain uptake than the other four subjects (Fig. 2b).

**Fig. 3:**
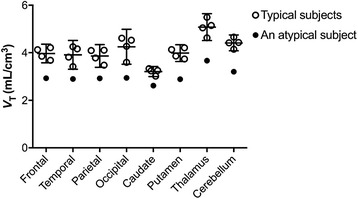
Comparison of the *V*_T_ for typical subjects and an atypical subject. Data for typical subjects represent the mean with the 95% confidential interval for four subjects

The preliminary kinetic analysis of the comparison of Akaike’s information criterion (AIC) (paired *t* test, *P* < 0.05) in all regions investigated showed that the two-tissue compartment model provided significantly better AIC scores than the one-tissue compartment model. The rank order of total distribution volume (*V*_T_) values (mL/cm^3^) of gray matter regions from the two-tissue compartment model (*n* = 4) was thalamus (5.1 ± 0.4) > cerebellum (4.4 ± 0.2) ≈ occipital cortex (4.3 ± 0.2) ≈ putamen (4.0 ± 0.2) ≈ frontal cortex (4.0 ± 0.2) ≈ temporal cortex (3.9 ± 0.4) ≈ parietal cortex (3.9 ± 0.3) > caudate (3.2 ± 0.1). One atypical subject showed significantly lower *V*_T_ than that of the four typical subjects (Fig. 3). However, the distribution pattern of radioactivity in the brain was similar among all five subjects.

### Metabolite analysis

**Fig. 4:**
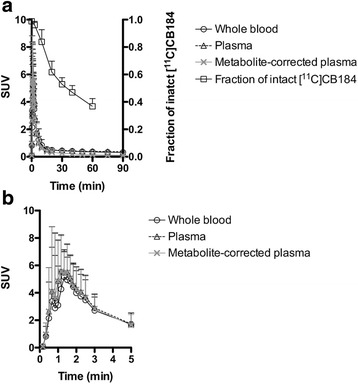
Mean decay-corrected TACs of whole blood, plasma, metabolite-corrected plasma, and fraction of intact [^11^C]CB184 after intravenous injection of [^11^C]CB184 into human subjects (**a**). Values for 5 min (**b**) were extracted from **a**. Data for the fraction of intact [^11^C]CB184 represent the mean ± SD of five subjects

Plasma radioactivity rapidly decreased after a bolus injection (Fig. 4a). The concentrations of radioactivity and the overall shapes of the TACs in blood and plasma were well matched (Fig. 4b). The results of high-performance liquid chromatography (HPLC) analysis of plasma are summarized in Table 1. We found no differences in the metabolite profile or plasma kinetics between the one atypical and four typical subjects.

**Table 1:** Percentages of radiolabeled metabolites in plasma after intravenous injection of [^11^C]CB184

Time (min)	HM1	HM2	HM3	[^11^C]CB184	LM1
3	1.4 ± 1.1	1.1 ± 0.7	0.3 ± 0.4	96.1 ± 2.7	1.2 ± 1.0
10	6.4 ± 3.7	8.7 ± 5.6	0.7 ± 0.7	83.8 ± 8.9	0.5 ± 0.6
20	12.2 ± 2.5	20.6 ± 6.0	5.0 ± 2.7	61.6 ± 8.0	0.6 ± 0.4
30	14.9 ± 2.5	25.3 ± 3.5	6.7 ± 3.7	52.9 ± 4.9	0.3 ± 0.3
40	18.4 ± 6.0	26.4 ± 4.8	7.7 ± 3.8	47.0 ± 5.0	0.5 ± 0.4
60	24.0 ± 8.1	29.4 ± 7.2	9.4 ± 3.3	36.7 ± 5.7	0.5 ± 0.7

Data are the mean ± SD for healthy male subjects (*n* = 5)

*HM* hydrophilic metabolite, *LM* lipophilic metabolite

The extraction ratio of plasma radioactivity into acetonitrile was >93%. In HPLC analysis, the recovery in the eluate was quantitative. [^11^C]CB184 was eluted at a retention time of 8.3 min. Three hydrophilic metabolites (HM1, 3.2 min; HM2, 4.6 min; and HM3, 6.4 min) and a lipophilic metabolite (LM1, 9.7 min) were detected. At 60 min after injection, [^11^C]CB184 was still the main compound detected (36.7% ± 5.7%, *n* = 5). The mean radioactivity voided into urine at 111 ± 17 min (range, 97–132; *n* = 8) was 0.3% ± 0.0% of the injected activity (range, 0.2–0.3, *n* = 8). In urine, broad hydrophilic metabolites that eluted between the elution front (2.6 min) and 6.4 min on the chromatogram were dominant (99.4% ± 0.6%, *n* = 3). The parent radioligand was not detected in voided urine.

### Whole-body imaging

**Fig. 5:**
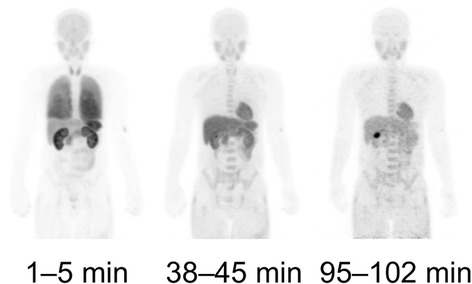
Representative whole-body decay-corrected maximum-intensity-projection images of [^11^C]CB184. Images were obtained at 1–5, 38–45, and 95–102 min after intravenous injection of [^11^C]CB184 into a healthy male subject

The representative whole-body distribution of [^11^C]CB184 in one subject is shown in Fig. 5.

**Fig. 6:**
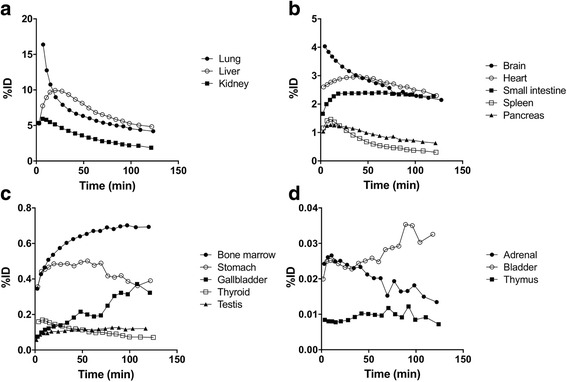
Regional decay-corrected TACs of 16 source organs (**a**-**d**) after intravenous injection of [^11^C]CB184 into the same subject as shown in Fig. 5. TACs were expressed as percent of injected dose (%ID). Each panels showed the TACs of source organs with high (**a**), moderate (**b**), low (**c**) and very low (**d**) radioactivity. Activities in bone marrow were estimated from thoracic and lumbar vertebrae. The initial time point for the lungs (25%ID at 3.3 min) was deleted because this high value unnecessarily extended the *y*-axis

Figure 6 shows the decay-corrected TACs of source organs for the same subject. The distribution of [^11^C]CB184 was consistent with the expected known distribution of TSPO in the body and was similar to that of other radioligands for TSPO [23–25].

The lungs had the highest uptake (25% injected activity) in the first frame. Uptake in the lung decreased thereafter however still dominant (4.2% injected activity) until the last frame (Fig. 6a), which reflects the high density of TSPO. Other organs with high densities of TSPO including the kidneys (Fig. 6a) and heart wall (Fig. 6b) were visible and retained radioactivity. The brain, which had a low TSPO density, was clearly visualized in the early time course and indicates higher brain permeability of [^11^C]CB184 than in other organs (Fig. 6b). The radioactivity in liver peaked at 20 min and showed the highest radioactivity of all organs thereafter (Fig. 6a). The gallbladder was clearly visible, and the radioactivity gradually increased (Fig. 6c), illustrating the hepatobiliary excretion of radioactivity. The radioactivity in the urinary bladder was very low (Fig. 6d) and not visible at any time. The mean ± SD of radioactivity voided into urine at 132 ± 0 min (*n* = 3) was only 0.3% ± 0% (*n* = 3) of the injected activity.

The normalized number of disintegrations is shown in Additional file 1: Table S1, and the organ absorbed and effective doses are shown in Table 2.

**Table 2:** Organ absorbed doses

Organ	Absorbed dose (μGy/MBq)
Adrenals	4.6 ± 0.9
Brain	4.0 ± 0.3
Breasts	2.2 ± 0.0
Gallbladder wall	5.4 ± 0.4
Heart wall	12.8 ± 0.5
Kidneys	21.0 ± 0.5
Liver	8.3 ± 0.9
Lower large intestine wall	2.3 ± 0.1
Lungs	16.8 ± 2.7
Muscle	2.2 ± 0.1
Osteogenic cells	3.6 ± 0.1
Ovaries	2.5 ± 0.1
Pancreas	16.5 ± 2.2
Red marrow	3.2 ± 0.1
Skin	1.7 ± 0.1
Small intestine	5.4 ± 0.9
Spleen	16.6 ± 6.6
Stomach wall	4.5 ± 0.9
Testes	3.6 ± 0.7
Thymus	2.1 ± 0.0
Thyroid	12.0 ± 3.9
Upper large intestine wall	2.8 ± 0.1
Urinary bladder wall	2.1 ± 0.1
Uterus	2.5 ± 0.1
Total body	2.8 ± 0.0
Effective dose (μSv/MBq)	5.9 ± 0.6

Data are the mean ± SD for healthy male subjects (*n* = 3)

The highest absorbed dose was observed in the kidneys, followed by the lungs, spleen, and pancreas. The mean ± SD estimated effective dose was 5.9 ± 0.6 μSv/MBq.

## Discussion

This is the first clinical study to assess the safety, radiation dosimetry, and initial brain imaging of [^11^C]CB184 in a small number of healthy human subjects.

We found that [^11^C]CB184 was safe and well tolerated, with no adverse effects in the eight subjects included in this study. The radiation-absorbed doses were higher in the kidneys, lungs, spleen, pancreas, heart wall, thyroid, and liver than in the other organs studied but was nonetheless sufficiently low for clinical use. The individual organ and total-body doses associated to [^11^C]CB184 PET were comparable to other ^11^C-labeled TSPO ligands [23, 25, 26].

[^11^C]CB184 was distributed in the gray matter regions. The regional distribution of [^11^C]CB184 was consistent with the TSPO density in the healthy human brain. The localization of TSPO in the normal human brain has been demonstrated by in vitro autoradiographic studies with ^3^H-PK11195 [27]. The highest signal level of TSPO was observed in the thalamus, followed by the cerebellum and other brain regions. Furthermore, the *V*_T_ values in the gray matter and the regional distribution patterns of [^11^C]CB184 closely resembled those of recently developed TSPO ligands such as [^11^C]PBR28 [13] and [^11^C]DPA-713 [15] (Table 3). Importantly, inter-individual variations of *V*_T_ values were much smaller for [^11^C]CB184 than those of [^11^C]PBR28 and [^11^C]DPA-713. This small inter-individual variation of *V*_T_ might have benefits for clinical studies such as in statistical parametric mapping.

**Table 3:** Comparison of regional gray matter *V*_T_ of four TSPO ligands measured in human subjects

Ligand	Frontal cortex	Temporal cortex	Parietal cortex	Occipital cortex	Putamen	Caudate	Thalamus	Cerebellum
[^11^C]CB184	4.0 ± 0.2	3.9 ± 0.4	3.9 ± 0.3	4.3 ± 0.5	4.0 ± 0.2	3.2 ± 0.1	5.1 ± 0.4	4.4 ± 0.2
[^11^C]DPA-713 [15]	4.1 ± 0.8	–	3.7 ± 0.7	–	3.5 ± 0.6	3.1 ± 0.6	4.7 ± 0.9	3.9 ± 0.9
(*R*)-[^11^C]PK11195 [15]	0.3 ± 0.1	–	0.4 ± 0.1	–	0.3 ± 0.1	0.3 ± 0.1	0.4 ± 0.1	0.3 ± 0.1
[^11^C]PBR28 [13]	–	–	3.9 ± 1.1	–	–	3.3 ± 1.0	4.6 ± 1.6	4.1 ± 1.3

Data represent the mean ± SD for healthy typical male subjects (*n* = 4). Data for [^11^C]DPA-713, (*R*)-[^11^C]PK11195, and [^11^C]PBR28 were taken from previously published reports [13, 15]

Although the peripheral metabolism of [^11^C]CB184 was faster than that of (*R*)-[^11^C]PK11195 [15], *V*_T_ values of [^11^C]CB184 were 10 times higher than those of (*R*)-[^11^C]PK11195 [15]. This higher *V*_T_ of [^11^C]CB184 may be due to the 7.9 times higher affinity and lower lipophilicity of CB184 compared to (*R*)-PK11195 [19].

In this small number study, we observed unusually lower binding of [^11^C]CB184 in the entire brain. We found no differences in plasma input function between the unusually lower binding and typical binding. This finding may indicate mixed-binding affinity of [^11^C]CB184 to TSPO in humans, due to the presence of an rs6971 polymorphism in the gene encoding TSPO [28]. Although imidazopyridineacetamides, like [^11^C]CB184, have different structural skeletons from newly developed other TSPO ligands, recently published data indicate that the imidazopyridineacetamides, [^18^F]PBR111 [29] and [^123^I]CLINDE [30], show the influence of a genetic polymorphism on the TSPO binding. Our human data suggest an approximately 1.4-fold difference between atypical binding and typical binding. This small difference may indicate the difference between high-affinity binding and mixed-affinity binding. If so, the effect of a genetic polymorphism for [^11^C]CB184 binding may be slightly weaker than that of other second-generation TSPO ligands [28]. To clarify these points, future studies should be performed to determine whether significant differences are present in [^11^C]CB184 binding among different types of rs6971 polymorphisms. For this purpose, in vitro autoradiographic analyses in postmortem human brain [31] will be effective to prove the influence of rs6971 polymorphisms on the binding affinity of CB184.

**Fig. 7:**
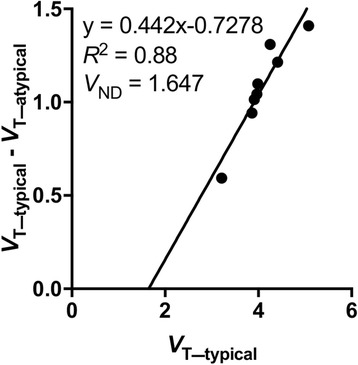
Estimation of *V*_ND_ of [^11^C]CB184. Polymorphism plot with *x*-intercept representing *V*_ND_. In this plot, the *x-axis* is the mean *V*_T_ for typical subjects and the *y-axis* is the difference between *V*_T_ for typical and atypical subjects

We used a two-tissue compartment model and calculated *V*_T_ as the outcome measure related to the cerebral TSPO density. To estimate the binding potential of [^11^C]CB184, a reference tissue model may be useful for quantification of TSPO. However, as shown in Fig. 3, the *V*_T_ in the entire brain region was decreased in an atypical subject. This indicates that specific binding is present in the entire brain and avoids the assumption of reference tissue modeling. Estimation of specific binding requires estimation of the non-displaceable volume of distribution (*V*_ND_) [32]. Estimation of *V*_ND_ requires a pharmacological blocking study [33]. If nonspecific binding (*V*_ND_) is homogeneous between subjects and within an individual subject, one can estimate the *V*_ND_ values by applying the polymorphism plot to the [^11^C]CB184 PET data across the population of high-affinity and mixed-affinity binding subjects [29]. We preliminary performed the polymorphism plot on the current data (Fig. 7). *V*_ND_ was estimated as the *x*-intercept value of 1.647.

The limitations of this study are the small sample size and lack of analysis of rs6971 polymorphisms. Further clinical studies with a larger sample size and genetic analysis are planned in our laboratory. The other limitation is the low density of TSPO in the healthy normal brain, which obscured confirmation of the sensitivity and specificity of [^11^C]CB184. Therefore, an additional study demonstrating strong TSPO expression in patients, such as glioma patients [34], is needed. The most critical comment is the short half-life of ^11^C, which considerably complicates the widespread use of [^11^C]CB184. The benefit of using ^11^C-labeled tracers is their lower radiation burden when serial PET scans are performed in the same subject. Very recently, ^18^F-fluoroethyl derivatives of CB184 were synthesized and show promising properties for TSPO imaging to detect neuroinflammation [35].

## Conclusions

The initial findings of the present study in a small group of subjects indicated that [^11^C]CB184 PET is feasible for imaging TSPO expression in the brain with an acceptable radiation dose and pharmacological safety at the dose required for adequate PET imaging. The brain uptake of [^11^C]CB184 can be calculated as *V*_T_, which is an index of TSPO density. The *V*_T_ values of [^11^C]CB184 corresponded well to the estimated TSPO density in the healthy human brain. In this small group analysis, we experienced unusually lower uptake of [^11^C]CB184, which may suggest the effect of a genetic polymorphism on the binding of [^11^C]CB184 to TSPO.

## Methods

### Subjects

All experiments were approved by the Tokyo Metropolitan Institute of Gerontology institutional review board (IRB) and were performed in accordance with the IRB rules and policies. All subjects gave study-specific informed consent to participate in the study, and all experiments were carried out in accordance with the relevant guidelines. The study was registered in UMIN-CTR (UMIN000020139) on December 9, 2015.

Eight healthy male subjects, aged 22–34 years (mean age ± SD, 26 ± 4 years), were enrolled in this study. The subject inclusion criteria included age between 20 and 60 years old, male, the ability to provide informed consent, and normal medical history, physical examination and vital-sign findings. The subject exclusion criteria included who has dysfunction in the liver and kidneys, abnormal findings in the CNS, cardiac failure, history of drug or food allergy, and judged by the clinical investigator to be inappropriate as a participant in this study. Five of the eight subjects were recruited into a dynamic brain PET study. The subjects weighed 50.1–70.6 kg (mean weight ± SD, 64.6 ± 8.8 kg). For anatomical co-registration, a three-dimensional (3D) fast spoiled gradient-echo (repetition time = 7.6 ms, echo time = 3.1 ms, inversion time = 400 ms, matrix = 256 × 256 × 196 voxels) T1-weighted whole-brain image was acquired for each subject on a GE Discovery MR750w 3.0T scanner (GE Healthcare, Wauwatosa, WI). The other three subjects participated in a whole-body distribution study. The subjects weighed 59.7–84.4 kg (mean weight ± SD, 69.2 ± 13.3 kg). All eight subjects were free of somatic and neuropsychiatric illnesses according to their medical history and findings of physical examination and had no brain abnormalities on MRI.

### Radiotracers

[^11^C]CB184 was prepared by *O*-methylation of the corresponding desmethyl precursor using [^11^C]methyl triflate as described previously [21].

### Safety monitoring

Safety data were collected after administration of [^11^C]CB184 and throughout the follow-up period of 1 week in five subjects. Safety monitoring included the recording of adverse events, changes in vital signs, physical examination, electrocardiogram, and laboratory parameters (serum biochemistry and hematology analysis). The detailed protocol for investigating safety monitoring was the same as that reported previously [36].

### Brain PET scanning

PET scanning was performed using a Discovery PET/computed tomography (CT) 710 scanner (GE Healthcare, Milwaukee, WI) in 3D mode. This scanner has an axial field of view of 15.7 cm, a spatial resolution of 4.5 mm full width at half maximum (FWHM), and a *Z*-axis resolution of 4.8 mm FWHM. We acquired 47 slices. After low-dose computed tomography (LD-CT) scanning to correct for attenuation, [^11^C]CB184 (609 ± 117 MBq/12.1 ± 6.1 nmol) was injected into the antecubital vein of each subject as a bolus for 1 min, and a 90-min dynamic scan (20 s × three frames, 30 s × three frames, 60 s × five frames, 150 s × five frames, and 300 s × 14 frames) was performed. Arterial blood (0.5 mL each) was sampled at 10, 20, 30, 40, 50, 60, 70, 80, 90, 100, 110, 120, 135, 150, and 180 s, as well as at 5, 7, 10, 15, 20, 30, 40, 50, 60, 75, and 90 min. The whole blood and separated plasma were weighed, and radioactivity was measured with a NaI (Tl) well scintillation counter (BeWell Model-QS03 F/B; Molecular Imaging Labo, Suita, Japan). To analyze the labeled metabolites, 1.5 mL additional blood was obtained at 3, 10, 20, 30, 40, and 60 min. After the PET scan, urine was obtained from each subject, and radioactivity was measured. Unaltered [^11^C]CB184 in the plasma was analyzed with HPLC, and the metabolite-corrected TAC of plasma was obtained as described previously [21].

Tomographic images were reconstructed using a 3D-ordered subset expectation maximization algorithm (subset, 16; iteration, 4) with incorporated time-of-flight information. The dynamic images were post-smoothed with a 4-mm FWHM Gaussian filter. The data were reconstructed in 128 × 128 × 47 voxels, and the voxel size was 2 × 2 × 3.27 mm. Partially overlapping circular regions of interests (ROIs) that were 10 mm in diameter were placed on the frontal, temporal, parietal, occipital, and cerebellar cortices, thalamus, putamen, and head of the caudate nucleus with reference to the co-registered MRI. TACs for these ROIs were calculated as becquerel per milliliter or as standardized uptake value (SUV): (activity/ml tissue)/(injected activity/g body weight). Using the TACs of tissues and the metabolite-corrected TAC of plasma, the *V*_T_ (*K*_1_/*k*_2_ × (1 + *k*_3_/*k*_4_)) for [^11^C]CB184 was evaluated using the one- and two-tissue compartment models. The goodness of fit by the two-model analysis was evaluated using AIC.

### Whole-body imaging

The protocol for investigating radiation dosimetry in human subjects using whole-body imaging was essentially the same as that reported previously [37].

Whole-body PET/CT scans were obtained using a Discovery 710 PET/CT scanner (GE Healthcare) in 3D mode. LD-CT was used for attenuation correction of the PET emission scan. The first PET acquisition was started 1 min after the intravenous bolus injection of 763 ± 40 MBq (9.9 ± 1.9 nmol) of [^11^C]CB184. Then, 128-min scans (18 frames, 13 bed positions per frame, overlap of 23 of 47 slices per bed, 15 s/bed × four frames, 30 s/bed × 12 frames, and 60 s/bed × two frames) from the top of the head to mid-thigh were performed. Images were reconstructed using a 3D-ordered subset expectation maximization algorithm (subset, 24; iteration, 2) with a 6.4-mm Gaussian filter. The recovery of radioactivity in whole-body PET/CT scans (total activity in the image/injected radioactivity) was quantitative at the first frame (89% ± 8%, at 1–5 min after injection, *n* = 3) and gradually only a little decreased to the last frame (76% ± 5%, at 115–128 min after injection, *n* = 3).

ROIs were manually placed over 16 organs that could be identified on PET or LD-CT: adrenals, brain, gallbladder, small intestine, stomach, heart wall, kidneys, liver, lungs, pancreas, bone marrow (thoracic and lumbar vertebrae), spleen, testes, thymus, thyroid, and urinary bladder. The decay-uncorrected and decay-corrected TACs of organs were calculated as the percent injected dose (%ID) per milliliter and the %ID per organ. The volume of bone marrow, in which only part of the organ could be measured, was substituted by the volume that was calculated from the mass of red marrow in the adult male phantom (1.12 kg for 73.7 kg of body weight) adjusted by the subject’s body weight and 1.04 g/mL as the specific gravity [38]. The normalized number of disintegrations (MBq-h/MBq administered) for each source organ is equal to the area under the time course of decay-uncorrected curve (%ID/mL) multiplied by the volume of the organ ROI. The area under the time course curve was calculated by summing the area from time zero to the endpoint of the scan and the area from the endpoint of the scan to infinity. The former area was calculated by trapezoidal integration. The latter area was calculated by integration of radioactive decay from the endpoint.

The absorbed doses in 25 target organs of the adult male phantom were estimated from the normalized number of disintegrations of source organs by implementing the Medical Internal Radiation Dose method using OLINDA/EXM (Vanderbilt University) [39]. The effective dose was also calculated by OLINDA/EXM using the methodology described in International Commission on Radiological Protection Publication 60 [40].

## Additional file

Additional file 1: Table S1. Normalized number of disintegrations calculated from whole-body [^11^C]CB184 PET in human subjects. (DOC 37 kb)

## Abbreviations

%ID: Percent injected dose
(*R*)-[^11^C]PK11195: (*R*)-*N*-(sec-butyl)-4-(2-chlorophenyl)-*N*-^11^C-methyl-2-naphthamide
[^11^C]CB184: *N*,*N*-di-*n*-propyl-2-[2-(4-[^11^C]methoxyphenyl)-6,8-dichloroimidazol[1,2-a]pyridine-3-yl]acetamide
3D: Three-dimensional
AIC: Akaike’s information criteria
CNS: Central nervous system
CT: Computed tomography
FWHM: Full width at half maximum
HM: Hydrophilic metabolite
HPLC: High-performance liquid chromatography
IRB: Institutional review board
LD-CT: Low-dose computed tomography
LM: Lipophilic metabolite
MRI: Magnetic resonance imaging
PET: Positron emission tomography
ROIs: Regions of interests
SD: Standard deviation
SUV: Standardized uptake value
TACs: Time activity curves
TSPO: 18-kD Translocator protein
*V*_ND_: Non-displaceable volume of distribution
*V*_T_: Total distribution volume
