# Supplementary material for: Assessment of safety, efficacy, and dosimetry of a novel 18-kDa translocator protein ligand, [11C]CB184, in healthy human volunteers
Source: EJNMMI Res. 2017 Mar 23;7:26. doi: 10.1186/s13550-017-0271-6 (PMC5364125; doi:10.1186/s13550-017-0271-6)
Supplement: Additional file 1: Table S1. — Normalized number of disintegrations calculated from whole-body [11C]CB184 PET in human subjects. (DOC 37 kb) [file 13550_2017_271_MOESM1_ESM.doc]

**Table S1.** Normalized number of disintegrations calculated from whole-body [^11^C]CB184 PET in human subjects

| Organ | Normalized number of disintegrations (MBq-h/MBq administered) |
| --- | --- |
| Adrenals | 1.3E-04 ± 5.7E-05 (9.5E-05 – 2.0E-04) |
| Brain | 1.6E-02 ± 1.3E-03 (1.5E-02 – 1.7E-02) |
| Gallbladder contents | 8.6E-04 ± 2.3E-04 (7.1E-04 – 1.1E-03) |
| Small intestine | 1.0E-02 ± 2.9E-03 (6.9E-03 – 1.3E-02) |
| Stomach | 3.2E-03 ± 1.7E-03 (2.1E-03 – 5.2E-03) |
| Heart wall | 1.3E-02 ± 6.2E-04 (1.3E-02 – 1.4E-02) |
| Kidneys | 2.2E-02 ± 4.7E-04 (2.2E-02 – 2.2E-02) |
| Liver | 4.2E-02 ± 5.4E-03 (3.8E-02 – 4.8E-02) |
| Lungs | 6.2E-02 ± 1.1E-02 (5.1E-02 – 7.2E-02) |
| Pancreas | 5.2E-03 ± 7.6E-04 (4.5E-03 – 6.0E-03) |
| Red marrow | 1.0E-02 ± 1.7E-03 (8.8E-03 – 1.2E-02) |
| Spleen | 1.0E-02 ± 4.5E-03 (4.9E-03 – 1.3E-02) |
| Testes | 4.3E-04 ± 1.1E-04 (3.1E-04 – 5.0E-04) |
| Thymus | 3.8E-05 ± 1.8E-06 (3.7E-05 – 4.1E-05) |
| Thyroid | 9.4E-04 ± 3.4E-04 (6.3E-04 – 1.3E-03) |
| Urinary bladder contents | 9.8E-05 ± 3.7E-05 (5.5E-05 – 1.2E-04) |
| Remainder | 2.9E-01 ± 2.0E-02 (2.8E-01 – 3.2E-01) |
|  |  |
| Data are the means ± SD (range) for healthy male subjects (*n* = 3). | |
